# A Patient-Specific Digital Twin Model for Haptic-Based Dental Simulation: Development and Pilot Evaluation of Its Static Design

**DOI:** 10.3390/dj14100642

**Published:** 2026-10-05

**Authors:** Teodor Raul Constantin, Mihaela Pantea, Lucian Toma Ciocan, Robert Mihai Bacalu, Alexandru Mihai Micu, Andreea Cristiana Didilescu, Laura Iosif, Miruna Dinescu, Ana Maria Cristina Țâncu, Silviu Mirel Pițuru, Carlos M. Serrano, Marina Imre

**Affiliations:** 1 Faculty of Dentistry, “Carol Davila” University of Medicine and Pharmacy, 37 Dionisie Lupu Street, District 2, 020021 Bucharest, Romania; teodor-raul.constantin2022@stud.umfcd.ro (T.R.C.); robert-mihai.bacalu0720@stud.umfcd.ro (R.M.B.); alexandru-mihai.micu0720@stud.umfcd.ro (A.M.M.); 2 Discipline of Prosthodontics, Faculty of Dentistry, “Carol Davila” University of Medicine and Pharmacy, 37 Dionisie Lupu Street, District 2, 020021 Bucharest, Romania; laura.iosif@umfcd.ro (L.I.); miruna.dinescu@drd.umfcd.ro (M.D.); anamaria.tancu@umfcd.ro (A.M.C.Ț.); marina.imre@umfcd.ro (M.I.); 3 Discipline of Dental Prostheses Technology, Faculty of Dentistry, “Carol Davila” University of Medicine and Pharmacy, 37 Dionisie Lupu Street, District 2, 020021 Bucharest, Romania; 4 Discipline of Embryology and Microbiology, Faculty of Dentistry, “Carol Davila” University of Medicine and Pharmacy, 8 Eroii Sanitari Boulevard, 050474 Bucharest, Romania; andreea.didilescu@umfcd.ro; 5 Discipline of Organization, Professional Legislation and Dental Office Management, Faculty of Dentistry, “Carol Davila” University of Medicine and Pharmacy, 37 Dionisie Lupu Street, District 2, 020021 Bucharest, Romania; silviu.pituru@umfcd.ro; 6 Department of Digital Dentistry and Comprehensive Oral Care, Academic Centre for Dentistry Amsterdam (ACTA), Gustav Mahlerlaan 3004, 1081 LA Amsterdam, The Netherlands; c.serrano@acta.nl

**Keywords:** virtual reality, haptic technology, simulation training, digital twin, dental education, three-dimensional imaging, patient-specific modeling

## Abstract

**Purpose**: Haptic and mixed-reality (MR) simulation technologies are increasingly used in preclinical dental education to support psychomotor skill acquisition and to reduce early patient exposure. This technical feasibility study aimed to develop a patient-specific digital twin patient model integrated into an MR haptic-based dental simulator and to explore dental students’ perceptions of it through an exploratory post-workshop survey. **Materials and Methods**: A digital twin patient model was created by integrating intraoral, facial, and cone-beam computed tomography (CBCT) datasets, and was implemented within the SIMtoCARE Dente haptic-based dental simulator (SIMtoCARE B.V., Vreeland, The Netherlands). In this single-centre pilot study, students completed a structured questionnaire assessing usability, perceived fidelity, and curricular relevance on the same day, following a single faculty-led workshop. Of the 50 students who attended, 41 completed the questionnaire (response rate: 82%). **Results**: The proposed workflow enabled the creation and implementation of the digital twin patient model. Students reported high acceptance and perceived educational value, with 92.7% supporting curricular integration. Prior exposure to haptic systems did not differ across demographic groups. Fourth-year respondents showed significantly higher agreement with the item on complementarity with conventional training. **Conclusions**: The digital twin patient model was positively perceived by dental students in this single-centre pilot study. These findings reflect immediate self-reported perceptions and require validation in larger, comparative and longitudinal studies.

## 1. Introduction

The primary goal of medical education has long been to prepare future practitioners with the competencies required for proficient clinical performance, drawing on the pedagogical methods and technological resources available at each stage of educational advancement [1,2]. Consequently, sustained efforts have been devoted to enhancing and optimizing the mechanisms through which dental students develop preclinical skills, with the broader objective of cultivating highly competent clinicians [3,4]. Educational approaches have progressively shifted from traditional textbook-based learning to the use of plaster models, plastic casts, and, relatively more recently, 3D-printed models mounted into conventional phantom-head simulators [5,6,7].

One of the newest developments in this domain includes mixed-reality (MR) haptic-based dental simulators integrated with extended reality technologies, which modify users’ perceptions by incorporating digital elements [6]. This substantial progress is primarily driven by the distinctive demands of dental education, wherein trainees are required to perform invasive procedures on real patients under close supervision. Such expectations underscore the critical need to reduce the gap between preclinical training and clinical practice [6,8,9,10].

Another notable advancement in this domain is the introduction of the patient-centred virtual reality (PC-VR) concept [11], in which trainees performed dental procedures on intraoral scan-based simulations, enhancing preparedness for irreversible clinical procedures while mitigating risk of patient harm [11]. The present study advances this patient-centred VR concept precisely at the level of the underlying model: whereas the PC-VR module of Serrano et al. relied on a single-modality replica of the patient—an intraoral scan [11]—the present workflow integrates three imaging modalities, intraoral, facial, and CBCT, into a single, haptic-simulator-compatible model, reproducing the dentition, craniofacial skeleton, and facial soft tissues of one identified patient within a single coherent representation. For clarity, the following terminology is used consistently throughout. Virtual reality (VR) denotes a fully computer-generated three-dimensional environment that replaces the user’s visual field. Mixed reality (MR) denotes systems in which virtual content is spatially registered with, and manipulated within, the physical environment; in the present context, the virtual anatomy is co-located with physical, handpiece-shaped haptic instruments operated by the trainee. VR–haptic simulation denotes any virtual environment in which force feedback is delivered through such instrumented devices.

Relatively recent scientific evidence [12,13] suggests that haptic simulation technologies are generally well received by preclinical dental students and may demonstrate a potential ability to discriminate users based on their baseline manual dexterity; the authors suggest that this emerging digital modality could contribute to the acquisition, retention, and transferability of students’ psychomotor skills [12,13].

Furthermore, ethical considerations play an increasingly central role in contemporary discussions of preclinical dental education. From an ethical standpoint, permitting inexperienced trainees to undertake clinical procedures without prior demonstration of competence in simulated settings has become increasingly difficult to defend [8,14]. In this context, simulation-based mastery learning positions competence as a prerequisite for clinical participation rather than its byproduct [8,14], thereby ensuring that early patient encounters are conducted within a structured and adequately prepared clinical framework for both students and patients, rather than functioning as experimental experiences [9]. Other recent scientific evidence [15,16] underscores that MR haptic-based dental simulators deliver calibrated force feedback, real-time performance analytics, and standardized, repeatable scenarios. Accordingly, these systems create a structured interface between conceptual knowledge and clinical performance while minimizing variability and risk during early clinical training [15,16], thereby addressing the previously mentioned ethical considerations.

Nevertheless, despite considerable technological advancements, multiple challenges continue to hinder the effective integration of haptic and extended-reality systems into dental curricula [6,10,17]. Frequently reported limitations include the absence of standardized clinical performance metrics, limited access to analytical tools for objective assessment of student performance, and inadequate levels of fidelity within simulated environments [6,10,17].

Aligned with the ongoing evolution of technology-supported education, the present study aimed to develop a digital twin patient model suitable for integration into a haptic-based dental simulator (SIMtoCARE Dente, SIMtoCARE B.V., Vreeland, The Netherlands), specifically intended for undergraduate dental practical training. Additionally, this study assessed the perceptions of dental students at the Faculty of Dentistry in Bucharest, Romania, regarding this innovative educational approach. As mentioned above, this model advances the patient-centred VR (PC-VR) concept through the multimodal integration of intraoral, facial, and CBCT data into a single patient-specific representation. Throughout this manuscript, the term “digital twin patient model” is used in the sense established within the dental literature, denoting a detailed, patient-specific virtual model of an individual’s oral and craniofacial structures that can, in principle, be maintained through continuous, real-time data exchange with the physical patient [18]. The model described here represents the anatomical, structural instantiation of this concept: it reproduces the anatomy of one identified patient across three imaging modalities, but it is static and does not incorporate continuous bidirectional data exchange with the physical patient. The transition to a dynamically synchronized model is addressed as a direction for further development. The proposed approach enables dental students to virtually rehearse personalized clinical procedures prior to patient care, while simultaneously developing proficiency in accurate case documentation and the use of facial and intraoral scanners.

## 2. Materials and Methods

The study received approval from the Research Ethics Committee of “Carol Davila” University of Medicine and Pharmacy in Bucharest, Romania (Project ID: po-35-f-03; Protocol number: 10329; date of approval: 7 May 2025), and it was conducted in full compliance with the Declaration of Helsinki (1975, updated in 2013). Written informed consent was obtained from all individuals involved in the study, including the patient whose clinical imaging data were used to develop the digital twin patient model, as well as from the students who participated in the survey evaluating this educational approach. Written informed consent has been obtained from the participants to publish this paper. For the patient whose identifiable facial images appear in this manuscript, separate, personalized written informed consent was additionally obtained, specifically authorizing the publication of identifiable, non-anonymized facial imagery for scientific and educational purposes.

### 2.1. Developing the Digital Twin Patient Model

#### 2.1.1. Digital Data Acquisition

In order to generate the digital twin patient model, three datasets were required: an intraoral scan, a facial scan, and a Cone-Beam Computed Tomography (CBCT scan).

- Intraoral scanning

**Figure 1:**
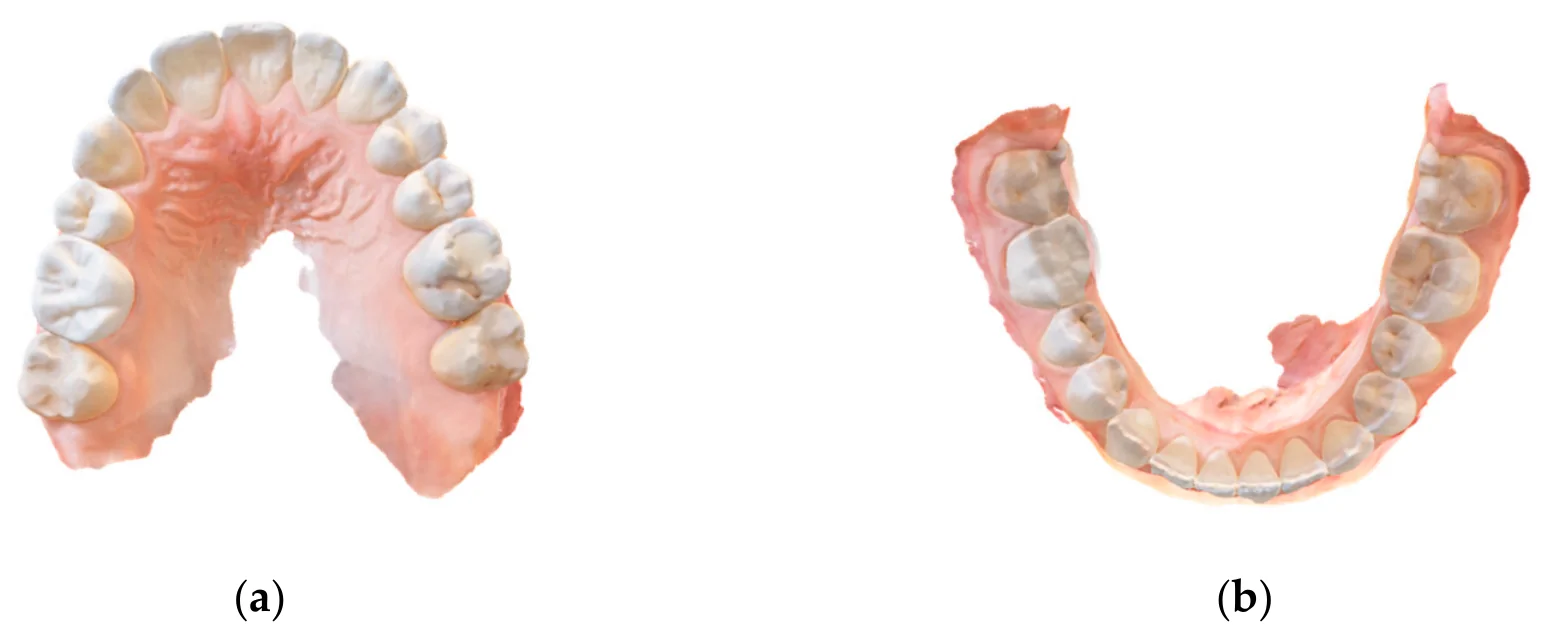
Obtained IO scans: (**a**) upper dental arch; (**b**) lower dental arch.

The intraoral scans were acquired using the Medit i700 scanner (Medit Corp., Seoul, South Korea) (Figure 1). Both maxillary and mandibular arches were captured with the assistance of a mouth retractor to ensure unobstructed access and optimal field of view. The raw datasets were processed in Meshmixer (Autodesk Inc., San Rafael, CA, USA) to remove artifacts and residual errors and to merge the data into a single watertight mesh, ensuring closed geometry suitable for further applications. The final models were exported in Polygon File Format (PLY), a widely used 3D data format that stores both geometry and colour information, enabling accurate representation and integration into subsequent workflows. PLY was chosen over the standard STL format because it retains texture and colour attributes in addition to geometry, which are essential for achieving an accurate visualization in MR–haptic environments.

B. CBCT data acquisition

**Figure 2:**
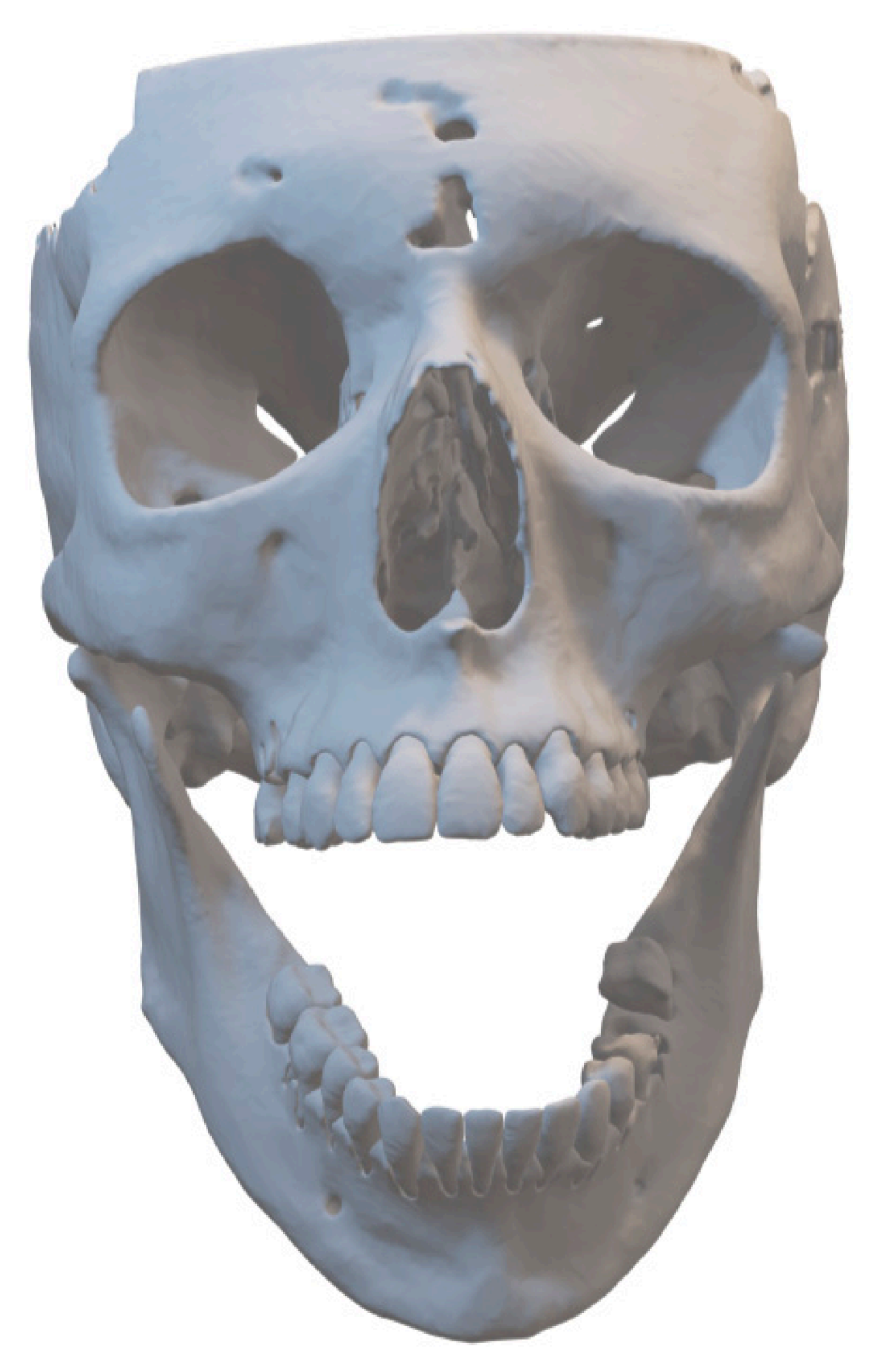
The obtained CBCT-scan.

In this study, a head CBCT examination was performed using a NewTom VGi evo unit (NewTom CONE BEAM 3D IMAGING, CEFLA S.C., Imola (BO), Italy), with the following exposure parameters: 110 kV, 4 mA, exposure time 3.5 s, voxel size 0.3 mm, field of view (FOV) 24 × 19 cm, and a dose–area product of 1687.5 mGy·cm^2^ (Figure 2). The resulting data were stored in Digital Imaging and Communications in Medicine (DICOM) format as volumetric datasets. Each voxel contains three-dimensional information, similar to a pixel in two-dimensional imaging. Segmentation was performed using BlueSkyBio (Blue Sky Bio LLC, Grayslake, IL, USA) to isolate relevant anatomical regions. The processed data were exported as stereolithography (STL) files, which describe the surface geometry of the segmented structures, enabling their integration with other three-dimensional datasets.

The CBCT dataset used for the construction of the patient-specific model was obtained from a single, clinically indicated examination performed on 15 December 2025, and was not acquired for research purposes alone. The patient presented a clinical indication for an open-mouth CBCT examination, established by the treating clinician in the context of the reported symptomatology—right-sided hemifacial pain, muscular tension, and chronic migraines, together with the clinical findings of right-sided temporomandibular joint (TMJ) pain on mandibular movement and articular sounds at the left TMJ. The examination was indicated to assess mandibular condylar translation and TMJ morphology, including the relationship between the mandibular condyle and the glenoid fossa/articular eminence, in the maximal mouth-opening position. Given this pre-existing clinical indication, the patient was invited to consider participation in the present study, on the basis that the clinically indicated imaging could also be used, with her consent, to construct the educational patient-specific model described in this manuscript. Prior to acquisition, the patient was informed that the open-mouth CBCT protocol would employ custom-manufactured devices designed to maintain the inter-arch (inter-occlusal) space during exposure, and she provided written informed consent authorizing the use of her imaging data for medical research and teaching purposes, including publication. This single acquisition simultaneously satisfied the clinical indication described above and provided the imaging dataset used for the construction of the model; no additional CBCT acquisition, and no additional radiation exposure, was performed for research or teaching purposes. The comparatively large field of view (24 × 19 cm) was clinically required rather than a smaller field limited to both temporomandibular joints: the presenting symptomatology—hemifacial pain, muscular tension, and chronic migraines—warranted assessment of the craniofacial skeleton more broadly, to identify or exclude other contributory skeletal or dental pathology (the patient had previously followed orthodontic treatment), and not only evaluation of the temporomandibular joints in isolation. The selected field of view therefore allowed simultaneous visualization of both temporomandibular joints (condyle–fossa complex, bilaterally) together with the wider maxillofacial skeleton within a single acquisition; its subsequent suitability for construction of the digital twin model was an incidental benefit of this clinically driven choice, rather than its rationale. The clinically required element of this protocol was the sustained maximal mouth-opening position during CBCT exposure, needed for assessment of condylar translation and joint morphology. To achieve and maintain this position reproducibly, custom, digitally designed and 3D-printed bilateral intraoral support devices (described in Section 2.1.1. C), dimensioned to the patient’s individually measured posterior interocclusal distance, were fabricated and used to maintain the mandible in the required opening position during CBCT exposure. The same devices were subsequently reused, following the CBCT examination, to reproduce a similar mouth-opening position during the facial scanning session—a level of precision and reproducibility that supported accurate multimodal registration of the digital twin model, extending beyond what the clinical examination alone would have required.

C. Facial scanning

**Figure 3:**
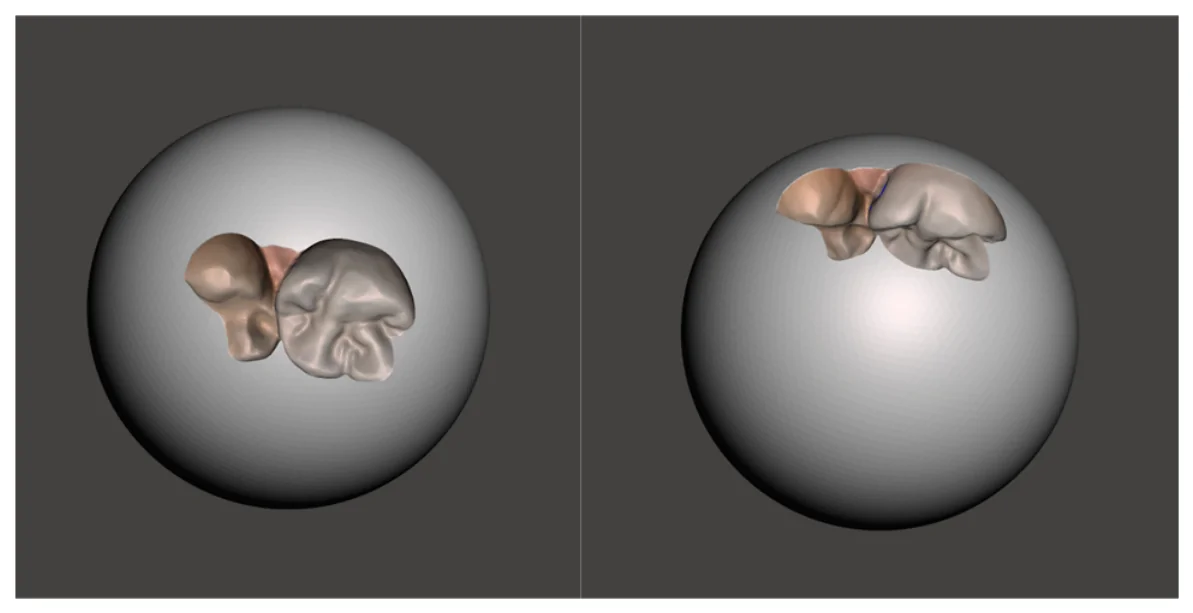
Digital project of the custom device designed to stabilize the dental arches at the predefined opening position (mean posterior interocclusal distance in unassisted maximal opening).

To ensure scan reproducibility and to allow the patient to keep the mouth open in a consistent open position during the procedure, a set of custom devices was fabricated (Figure 3) to stabilize the dental arches at a predefined opening position, set close to the patient’s maximal unassisted opening as determined prior to scanning. These devices, placed bilaterally between the upper and lower permanent first molars, functioned as intraoral supports, maintaining mouth opening and stabilizing the jaws throughout the scanning procedure. Prior to fabrication of these devices, the patient’s maximal unassisted mouth opening was measured clinically at two reference levels. Anteriorly, the vertical distance between the mid-incisal edge of the maxillary right central incisor (tooth 11) and the corresponding point on the antagonist mandibular incisor (tooth 41) was approximately 5.5 cm. Posteriorly, the vertical (interocclusal) distance between the buccal-mesial cusps of the maxillary and mandibular first molars (teeth 16 and 46) was measured bilaterally and averaged, yielding a mean posterior interocclusal distance of approximately 3 cm. The smaller value obtained posteriorly, relative to the anterior (incisal) measurement, is consistent with the rotational–translational biomechanics of mandibular opening: points located closer to the temporomandibular joint (hinge) axis, such as the first molars, describe a smaller arc of movement than points located further away, such as the incisors, for the same degree of condylar excursion. The 3 cm dimension therefore reflects the patient’s individually measured posterior interocclusal distance at maximal unassisted opening, rather than an arbitrary value, and was implemented as a 3 cm-diameter sphere designed in Meshmixer (Autodesk Inc., San Rafael, CA, USA) and adapted to the occlusal surfaces of the first molars bilaterally.

The devices were 3D-printed in NextDent SG (Surgical Guide) resin (3D Systems/NextDent B.V., Soesterberg, The Netherlands), a biocompatible, 405 nm photopolymer, post-cured according to the manufacturer’s instructions and disinfected prior to placement. Following fabrication, each device was clinically adapted intraorally: fit and stability were verified chairside and, where necessary, the devices were selectively adjusted/relieved using a straight handpiece with acrylic burs to achieve a stable, reproducible seating position on the occlusal surfaces of the first molars. The devices were retained passively and safely through firm, bilateral positioning between the maxillary and mandibular first molars, exploiting the stability provided by simultaneous contact with the opposing occlusal surfaces on both sides of the arch; no active fixation or adhesive was required. The risk of posterior displacement or aspiration was mitigated by design: each device’s dimensions and bilateral, symmetric seating between opposing molar surfaces prevented posterior migration toward the oropharynx, and device position was continuously visually monitored throughout placement, scanning, and removal. Fit and comfort were assessed clinically in two stages: each device was first tested individually on each side, and subsequently both devices were tested together (bilaterally) to confirm a comfortable, stable, and symmetric opening. For this patient, no discomfort, instability, displacement, or other adverse event was observed or reported at any stage of device placement, scanning, or removal.

**Figure 4:**
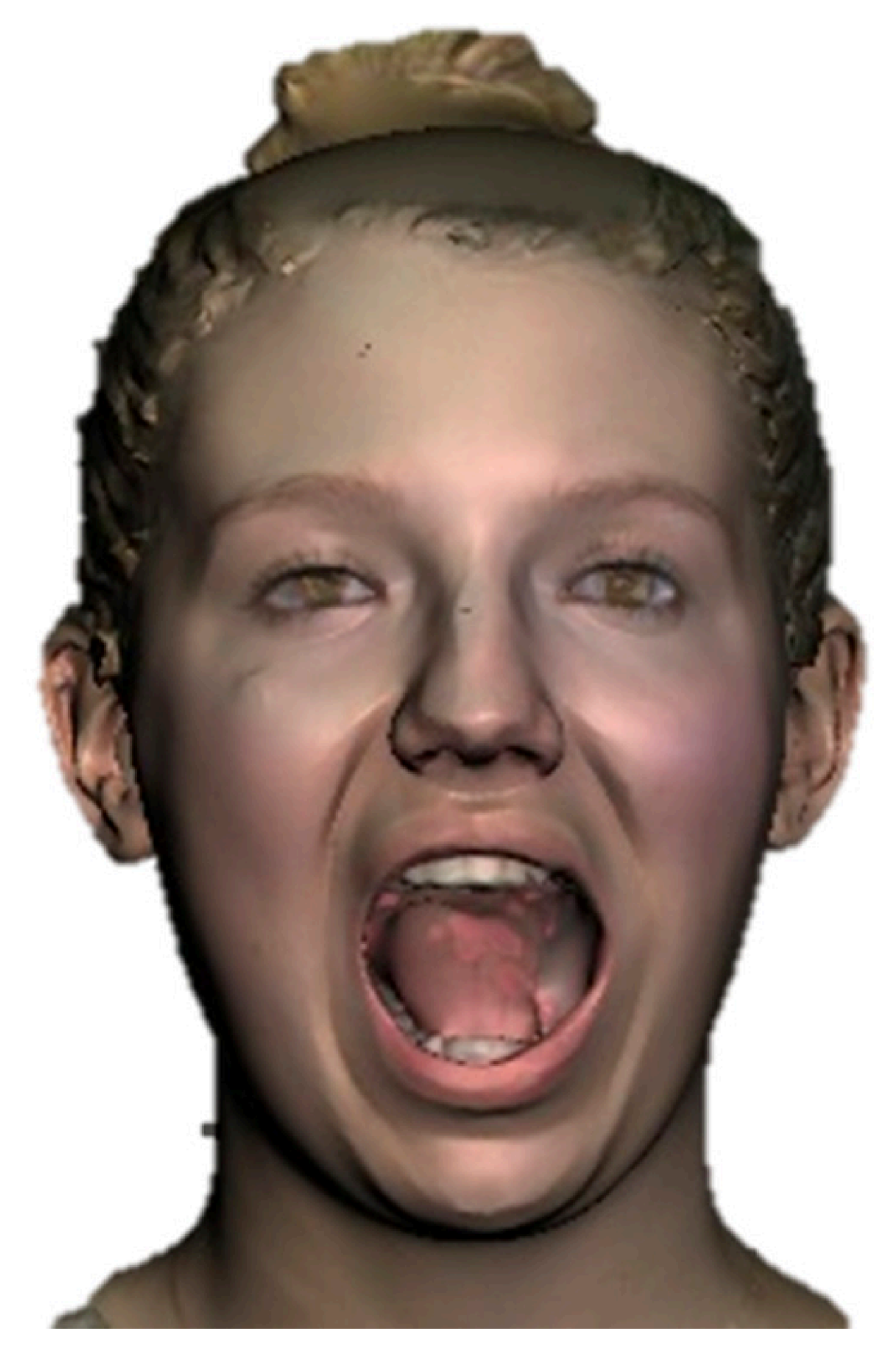
The obtained facial scan.

The facial scan (Figure 4) was acquired with the EinScan HX handheld 3D scanner (Shining 3D Tech. Co., Ltd., Hangzhou, China). For later alignment with intraoral and CBCT data, it was essential to capture the facial soft tissues and at least three teeth—one frontal tooth and one from each lateral side. The resulting dataset was exported in Polygon File Format (PLY) and then post-processed to remove artifacts and generate a single continuous mesh suitable for integration with other 3D datasets. Because the reconstructed facial soft-tissue model retains identifiable anatomical features, the facial dataset is not anonymized; its use and publication were authorized under the patient’s specific informed consent.

#### 2.1.2. The Superimposition of Digital Datasets

The registration and superimposition of the datasets were performed in Medit Design (Medit Corp., Seoul, Republic of Korea). Meshmixer (Autodesk Inc., San Rafael, CA, USA) was used for mesh post-processing only—removal of artefacts and residual errors, consolidation into a single watertight mesh merging of the raw scan data into a single watertight mesh, and design of the custom intraoral supports.

Landmarks and registration method: The three datasets (intraoral scan, facial scan, and segmented CBCT) were aligned in Medit Design (Medit Corp., Seoul, Republic of Korea) using anatomically consistent reference landmarks identifiable across at least two datasets. Specifically, the mesio-incisal angle of the upper right central incisor (tooth 11) and the buccal cusps of the maxillary second premolars were used as the primary registration landmarks. Corresponding landmarks were used for the mandible (mesio-incisal angle of the lower right central incisor (tooth 41) and the buccal cusps of the mandibular second premolars). The intraoral scans were first aligned to the STL files of the craniofacial skeleton using these dental landmarks, establishing a common dental-skeletal reference framework; the facial scan was then registered to this combined model using the same anatomical references (the frontal tooth and the bilateral posterior teeth captured during facial scanning), together with soft-tissue landmarks of the perioral region.

Mesh post-processing: Prior to registration, the raw intraoral and facial datasets were post-processed in Meshmixer (Autodesk Inc., San Rafael, CA, USA) to remove scanning artefacts and residual errors and to merge the data with closed geometry suitable for subsequent alignment. Meshmixer was also used to design the custom bilateral intraoral supports described in Section 2.1.1. C.

Software versions and settings: The following versions were in use at the time of model construction (March 2026): Registration and superimposition were performed in Medit Design v2.1 (Medit Corp., Seoul, Republic of Korea), running within Medit Link v3.4.9. Alignment was carried out by manual landmark-based pre-alignment on the reference points listed above, followed by best-fit surface registration. Mesh post-processing was performed in Meshmixer 3.5.474 (Autodesk Inc., San Rafael, CA, USA), the final released build of that application. CBCT segmentation was performed in BlueSkyPlan 5.0 (Blue Sky Bio LLC, Grayslake, IL, USA).

**Figure 5:**
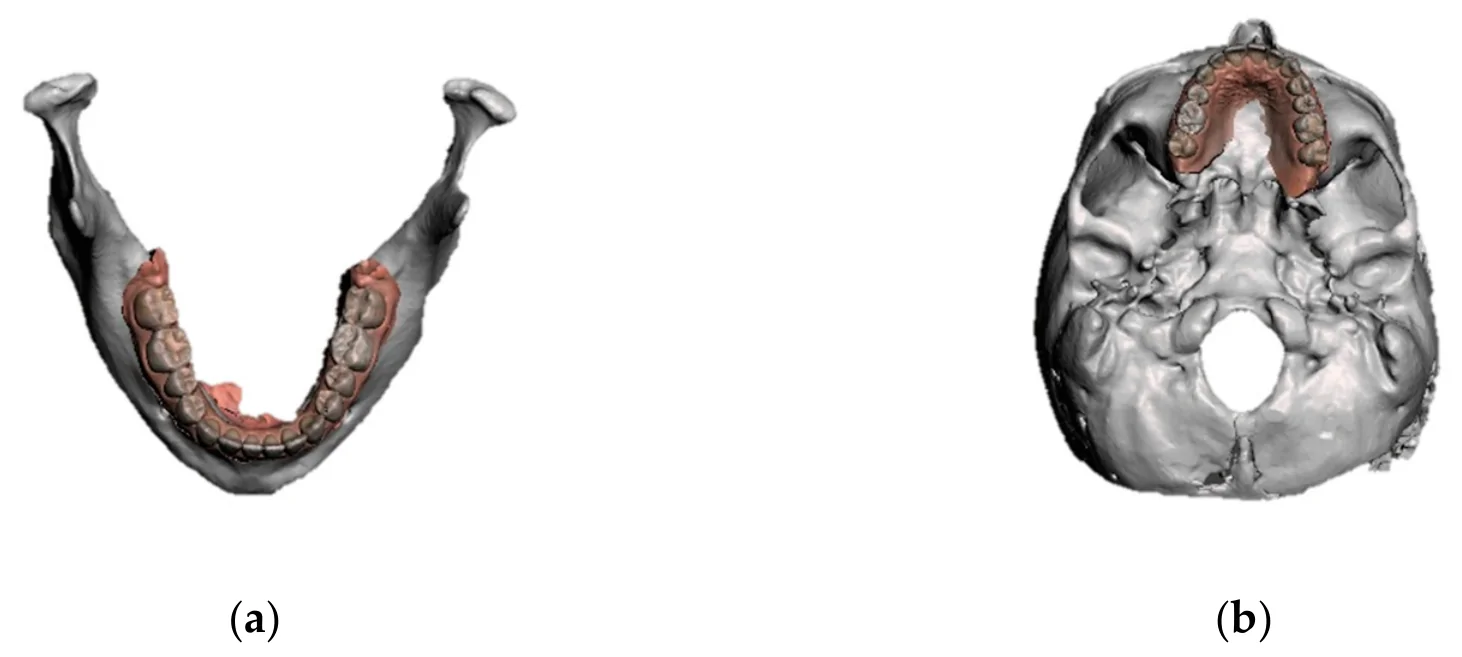
First superimposition step: (**a**) lower part; (**b**) upper part.

Initially, the integration process began by aligning the intraoral scans to the STL files of the craniofacial skeleton, which established correspondence between dental and skeletal structures (Figure 5).

**Figure 6:**
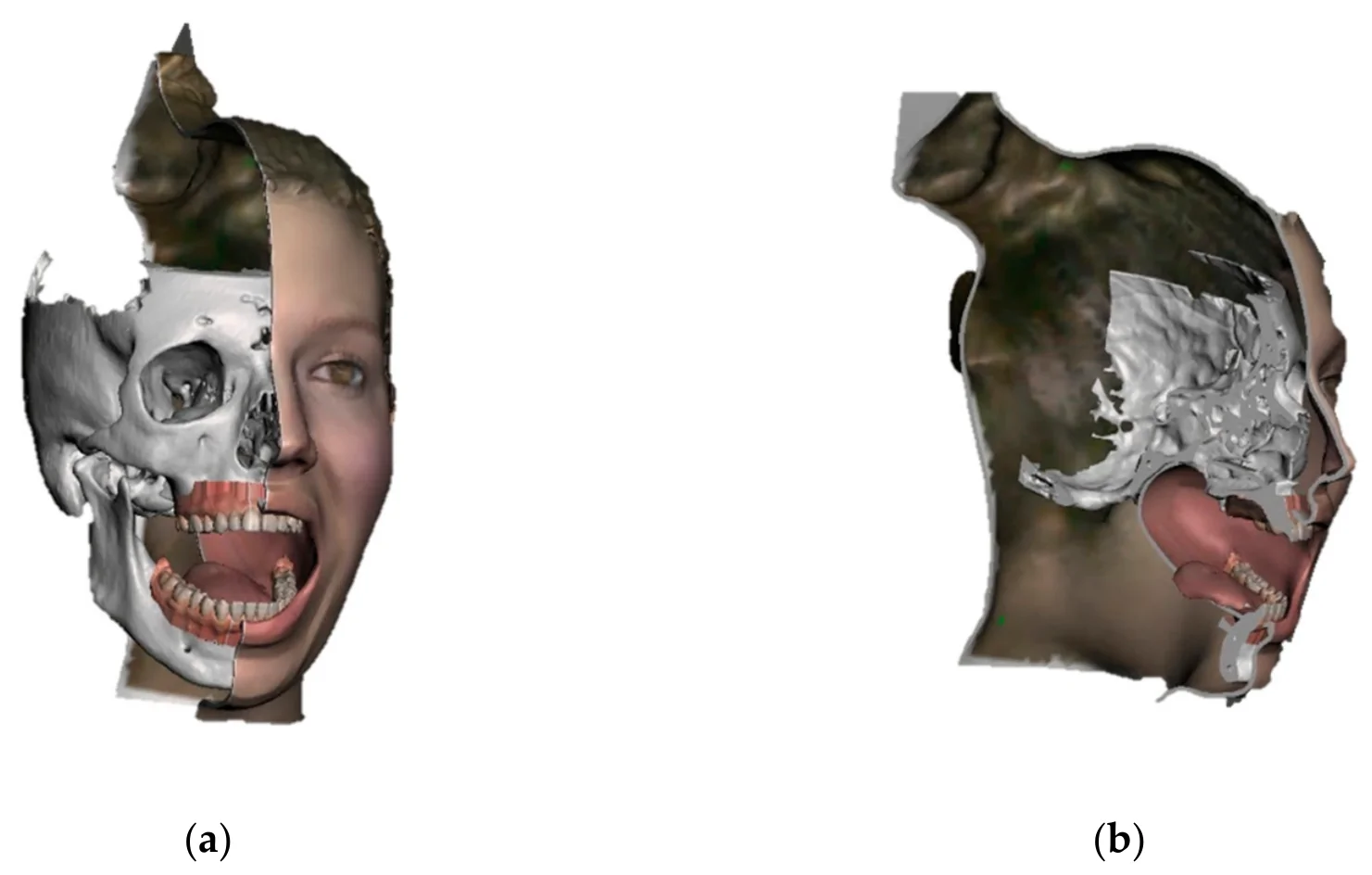
The final digital twin patient model: (**a**) front view; (**b**) sectional view.

The facial scan was registered to the combined craniofacial and dental model, obtaining the final integrated model (Figure 6). Finally, dental landmarks identified during facial scanning facilitated the integration of soft tissue morphology with the underlying hard tissue structures.

The internal file-conversion pipeline used to generate the SIMtoCARE-native import files, as well as the material and haptic parameters (e.g., stiffness, friction, and damping coefficients assigned to enamel, dentin, and soft tissue) applied within the SIMtoCARE Dente software (version 9021), are proprietary to the manufacturer, SIMtoCARE B.V., and are not disclosed to end users, including the authors; these parameters are therefore not reported in detail here.

Incorporating the facial scan was intended to achieve a multimodal patient-specific model integrating intraoral and skeletal datasets within the haptic environment. No objective, quantitative measure of registration accuracy (e.g., root-mean-square surface deviation between the intraoral, facial and CBCT-derived datasets) was computed for the present model; the integration of the three datasets is therefore described here in qualitative terms, and the resulting model should be regarded as a feasibility/proof-of-concept demonstration of multimodal patient-specific model construction, rather than a validated measure of anatomical accuracy.

Accordingly, the present model is patient-specific in geometry but not in haptic behaviour: because distinct material densities cannot currently be assigned to individual tissues, the force feedback re-flects the platform’s generic tissue presets rather than the individual mechanical properties of this patient’s dental and craniofacial tissues.

### 2.2. Evaluation of Undergraduate Students’ Opinion on the Use of the Digital Twin Patient Model in University Dental Education

#### 2.2.1. Survey Methodology

Students’ views on this proposed digital twin patient model were collected using a questionnaire distributed as a Google Form via email. Eligible participants were invited to complete the survey and were informed about the study in line with the principles of the World Medical Association Declaration of Helsinki and current European privacy regulations. At the beginning of the form, participants were provided with key details: the research objective, a note on the anonymity of the responses, and the assurance that they could withdraw at any point by stopping the questionnaire. Each invited student received an email containing written information about the study and the informed consent document. Informed consent was obtained from all participants prior to their involvement; consent was additionally confirmed upon submission of the completed questionnaire. In addition, informed consent for publication was also obtained. No personal or sensitive information was gathered, as the online survey was anonymous, and measures were in place to ensure that each participant could only complete the form once. Students were explicitly informed, both verbally and in writing, that participation was voluntary, optional, and would not affect their academic results. Responses were collected anonymously via Google Forms (Google LLC, Mountain View, CA, USA), configured to limit submissions to one response per participant while explicitly not collecting email addresses; no name, email address, or other direct identifier was collected at any stage. After data collection was complete, the 41 responses were assigned sequential identifiers (1–41) for organizational purposes during data entry and statistical analysis; this numbering was applied to the anonymous response data itself and was never linked to consent forms or to any identifying information. The investigators therefore had access only to anonymized responses at every stage of the study.

#### 2.2.2. Participant Selection

This pilot study was conducted among fourth-, fifth-, and sixth-year dental students enrolled at the Faculty of Dentistry, Carol Davila University of Medicine and Pharmacy.

Inclusion criteria required participants to be undergraduate students in the specified (clinical) years, aged 18 years or older, who had completed a full semester (14 weeks) of both theoretical (2 h/week) and practical (3 h/week) training in Fixed Prosthodontics. Practical training comprised the performance of diverse procedures on didactic dental models mounted in phantom heads and the performance of selected clinical procedures on patients. Additional inclusion criteria comprised completion of the practical and theoretical semester examination and participation in a workshop on haptic training using the proposed digital twin patient model. Students of any gender were eligible. Successful completion of the corresponding preclinical prosthodontics semester examination was required as an inclusion criterion because all participants performed the same standardized crown-preparation task within an individually allocated hands-on period; this ensured that every participant had already demonstrated the baseline theoretical and manual competence needed for the exercise to be meaningful and for exposure to be comparable across the cohort.

Exclusion criteria included failure to complete the semester-long practical/clinical training, absence from the practical and theoretical examinations, or refusal to participate. Students’ perceptions of the digital twin patient model and its potential use for training during university practical clinical training were assessed through an online questionnaire, as previously described. The study included fourth-year students, who were at the beginning of their clinical training with limited experience in performing clinical dental procedures, as well as fifth- and sixth-year students, who had intermediate to advanced clinical skills. Special emphasis was placed on obtaining their immediate feedback following the workshop.

No a priori sample size calculation was performed. As a pilot study, the number of places was determined by the capacity of the workshop: a single simulator unit was available for five hours per working day across ten working days, providing 50 h of individual hands-on time and therefore 50 places of one hour each. Recruitment was open to all eligible fourth-, fifth-, and sixth-year students through a single general announcement circulated by the Students’ League of Dental Medicine of Bucharest, and places were allocated on a first-come, first-served basis; as the invitation was not issued separately by year, the number of eligible students invited per year was not tracked. Of the 50 students who obtained a place and attended the workshop, 25 were fourth-year, 16 were fifth-year, and 9 were sixth-year students. All 50 attendees were invited to complete the questionnaire, and 41 did so (response rate: 82%), comprising 20 fourth-year, 12 fifth-year, and 9 sixth-year respondents. Age and gender were collected only through the questionnaire itself and are therefore not available for the 9 non-respondents; year of study, however, was recorded at registration for all 50 attendees and is reported above.

#### 2.2.3. The Haptic Workshop

Prior to the workshop, all registered students received an email describing the workshop and its objective, introducing a new preclinical training method using a digital twin patient model derived from a real patient, integrated into a haptic-based dental simulator, together with the written informed consent document for prior review.

Pre-session preparation: Before beginning the hands-on session each day, all participating students underwent a standardized 30-min preparatory session, during which attendance was recorded and they received information on occupational/laboratory safety rules together with a standardized explanation of how the haptic-based dental simulator functions and of the clinical task to be performed.

Simulator availability and hands-on time: A single haptic simulator unit (SIMtoCARE Dente) was used for all sessions. The workshop was scheduled over a two-week period (10 working days), from 2 to 15 March 2026, with five hours of simulator availability per working day (50 h in total), which provided one hour of individual, supervised hands-on time on the simulator for each of the 50 participating students. As mentioned, an additional 30 min per day was dedicated to introductory explanation and preparation before hands-on use of the haptic device.

Supervision and standardization: Four organizers/instructors (members of the research/teaching team) were present each day throughout the workshop to supervise students and standardize instructions. All students performed an identical, standardized clinical task on the digital twin model—preparation of tooth 11 (upper right central incisor) for a full-ceramic crown restoration, using a high-speed handpiece with diamond burs—under direct, continuous supervision and guidance. Because the task, instructions, supervision protocol and duration were identical for every participant, exposure to the exercise was standardized across the cohort. The procedure consisted of a full-coverage tooth preparation for an all-ceramic crown on tooth 11, comprising circumferential reduction in the labial, palatal, and proximal surfaces together with incisal reduction, rounded internal line angles to minimize stress concentration within the ceramic restoration, and a rounded shoulder finish line (approximately 1.0 mm), placed supragingivally to accommodate the all-ceramic restoration.

Questionnaire timing: Following completion of the haptic hands-on session, the structured questionnaire was distributed to each student by email; students were asked to complete it as soon as possible, on the same day as their participation in the workshop, in order to capture their immediate post-exposure perceptions.

#### 2.2.4. Survey Questionnaire

The questionnaire comprised four items addressing general aspects: age, gender, and year of study, along with one item assessing students’ previous experience with VR or haptic simulators; and 12 Likert-type items (rated on a scale from 1 = strongly disagree to 5 = strongly agree) designed to evaluate students’ perceptions of the learning method using the proposed digital twin patient model.

To facilitate the presentation and discussion of results, the 12 items were grouped into three descriptive, face-valid thematic categories; these groupings were not derived from, or tested using, formal psychometric procedures such as factor analysis, and no composite domain scores were computed or analyzed. All statistical analyses in this study were conducted at the level of individual items. The first category (*Model quality and anatomical fidelity*—Items #1, 2, 3, 8, and 9) captured students’ perceptions of anatomical fidelity, visual accuracy, and the overall realism of the proposed digital twin patient model, including the integration of multiple data sources such as facial scans and CBCT scans. It also enabled comparisons with conventional phantom-head simulators. The second category (*Educational value*—Items #4, 5, 6, and 7) highlighted how the proposed simulation may prepare students for future clinical, irreversible procedures. From a psychological perspective, it also emphasized stress reduction, increased confidence, and the development of a responsible, patient-centred clinical attitude in a supportive learning environment. The third category (*Usability, curricular value, and educational integration*—Items #10, 11, and 12) focused on the system’s user-friendliness, students’ openness to integrating this learning method into the preclinical curriculum, and its broader training value. Taken together, these three domains reflect the key characteristics of the developed model: fidelity, educational impact, and curricular relevance. Questionnaire items were deliberately presented in a non-grouped (shuffled) sequence to reduce pattern-based responding. This measure does not, however, constitute evidence of the instrument’s psychometric validity or reliability.

The English and Romanian versions of the questionnaire were developed in parallel by the bilingual members of the research team, rather than through a formal forward-translation/back-translation procedure. Linguistic and conceptual equivalence between the two versions was reviewed by all team members and subsequently assessed for clarity by five bilingual doctoral dental students, with advanced proficiency in English; no substantial modifications were required. We acknowledge that this represents a pragmatic, face-validity-based approach to bilingual adaptation rather than a formally validated translation methodology. The Romanian-language version administered to participants is provided in the Supplementary Materials alongside the English version. The present investigation may be considered a preliminary exploratory application of this instrument. As previously stated, the final version of the questionnaire was approved by the Scientific Research Ethics Committee of “Carol Davila” University of Medicine and Pharmacy, Bucharest, Romania (Project ID: po-35-f-03; Protocol No.: 10329; approval date: 7 May 2025).

#### 2.2.5. Data Analysis

All the data from the study were analyzed using IBM SPSS Statistics 25 (IBM Corp., Armonk, NY, USA) and illustrated using Microsoft Office Excel/Word 2024 (Microsoft Corp., Redmond, WA, USA). Qualitative variables were written as counts or percentages. Differences between groups were tested using Fisher’s Exact Test. Z-tests with Bonferroni correction were used to further detail the results obtained in the contingency tables. Implementation of the Benjamini–Hochberg method for FDR adjustment of item comparison according to demographic factors was done in R software (The R Project for Statistical Computing version 4.4.0). The Benjamini–Hochberg correction was applied separately within each demographic family of comparisons—the 12 item × age comparisons, the 12 item × gender comparisons, and the 12 item × study-year comparisons—each family therefore comprising 12 tests; no further adjustment was applied across the three families. Where the omnibus Fisher’s Exact Test indicated a significant difference, the z-tests of column proportions with Bonferroni correction described above were used as post hoc comparisons to identify which response categories differed between groups. The threshold considered for the significance level for all tests was considered to be *α* = 0.05.

## 3. Results

### 3.1. The Resulting Digital Twin Patient Model

Through the superimposition of the three datasets, a digital twin patient model was created, which was subsequently prepared for integration into the SIMtoCARE platform and transferred to their haptic-based dental simulator. In order to do that, we had to import two models: “upper part” and “lower part”. The “upper part” was represented by the .Ply file with the cranial structure, combined with the upper intraoral scan, while the “lower part” was represented by the facial scan file combined with the lower intraoral scan. The designations “upper part” and “lower part” are those used by the SIMtoCARE Dente software for the two mesh files it accepts on import, rather than anatomical descriptors; the datasets were therefore distributed between these two files so that each contained one dental arch, with the craniofacial skeleton combined with the maxillary intraoral scan and the facial soft-tissue shell combined with the mandibular intraoral scan.

**Figure 7:**
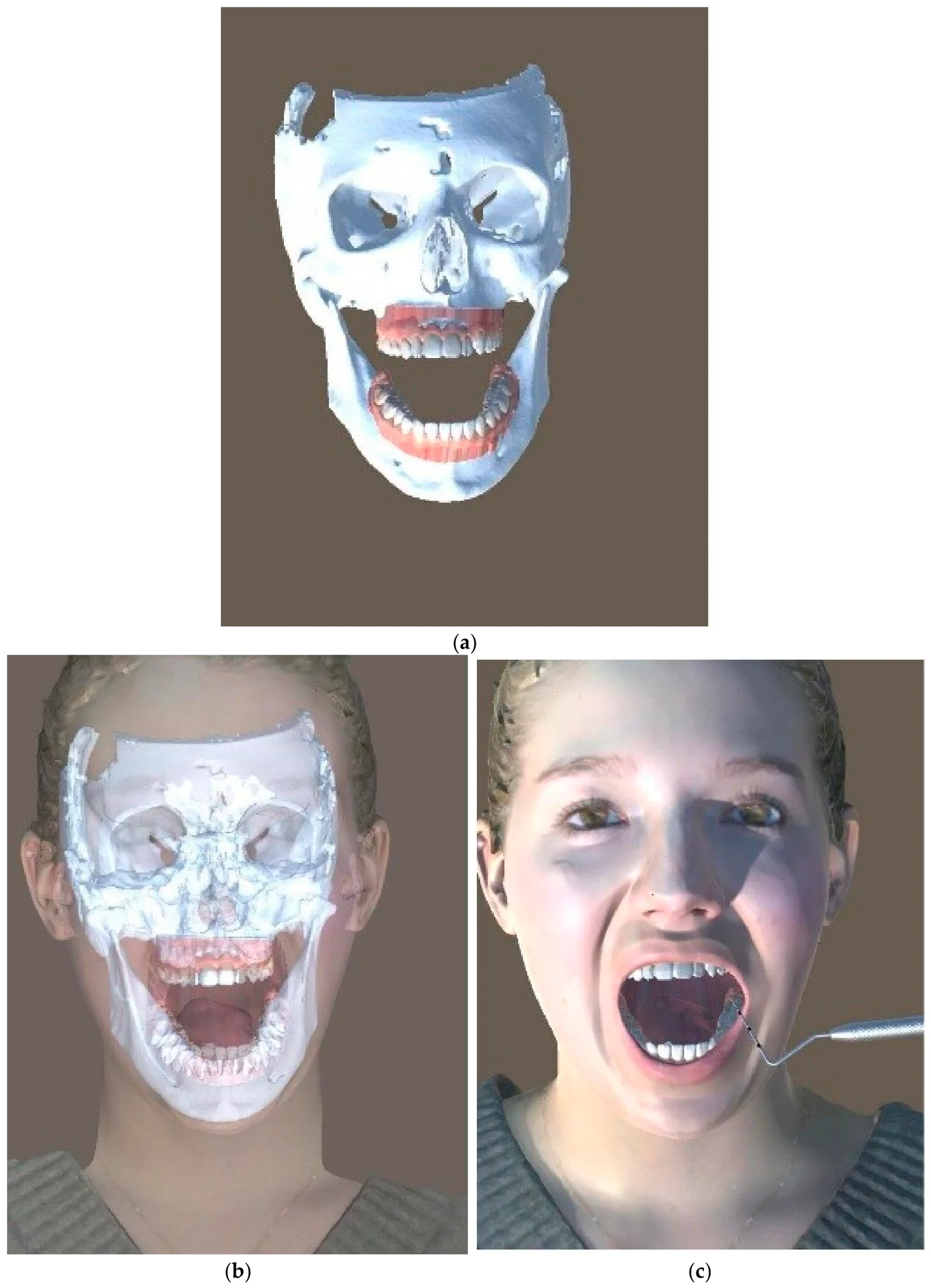
The digital twin patient model integrated into the haptic-based dental simulator: (**a**) upper and lower arches; (**b**) full translucent model; (**c**) full model for examination.

When performed as a dedicated protocol, the procedures required to construct the patient-specific model—a CBCT examination, facial scanning, and intraoral scanning—together with the subsequent digital processing steps, can together be completed within approximately two days. In the present case, the CBCT dataset originated from the patient’s pre-existing clinical examination, and the facial and intraoral scanning session was performed in a separate, subsequent session, with the remaining fully digital steps completed on the following day (Figure 7). This workflow allowed the creation of a patient-specific, multimodal digital twin patient model, which can be used by students to perform virtual examinations of the clinical case or to rehearse procedures prior to performing them on the real patient.

**Figure 8:**
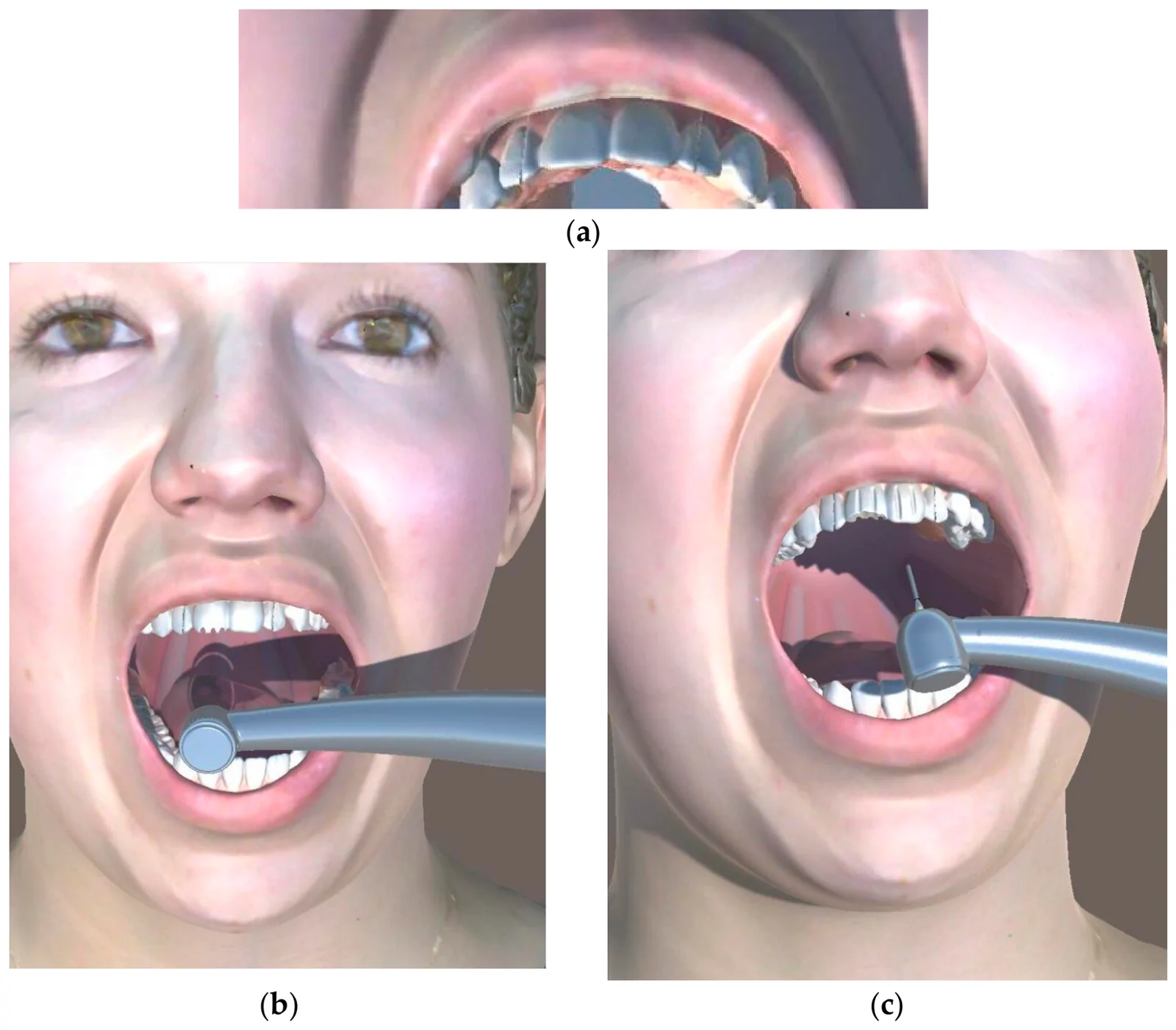
Steps of the prosthodontic training session performed on the digital twin patient model within the MR haptic-based dental simulator: (**a**) intraoral examination; (**b**) incisal reduction; (**c**) labial reduction. Published with the patient’s specific informed consent for the publication of identifiable facial imagery.

Under the supervision of the teaching staff, students performed prosthodontic procedures (e.g., tooth 11—tooth preparation for a full-ceramic crown) on the digital twin patient model. Students were also given the opportunity to repeat the virtual procedures over a period of one hour. In Figure 8, images from the prosthodontic practical training session using the digital twin patient model within the SIMtoCARE Dente MR haptic-based dental simulator are presented.

### 3.2. Survey Results: Students’ Opinion on the Use of the Digital Twin Patient Model

A total of 50 students attended the prosthodontic practical training workshop, which incorporated a digital twin patient model within an MR haptic-based dental simulator. All attendees were invited to take part in the survey, and 41 students voluntarily agreed, indicating a response rate of 82%. Each of the 41 respondents completed the questionnaire in full. To ensure data integrity, the survey platform was configured to permit only a single submission per participant.

Mean age was 23.15 ± 1.06 years (median = 23 years), most of the participants having 22 years (*n* = 14, 34.1%) or 23 years (*n* = 13, 31.7%). 24 participants (58.5%) were women, 20 participants (48.8%) were enrolled in the 4th university year, 12 participants (29.3%) were enrolled in the 5th university year and 9 participants (22%) were enrolled in the 6th university year. According to previous experience with VR or haptic simulators, 14 participants (34.1%) had no experience, 16 participants (39%) had limited experience, 8 participants (19.5%) had moderate experience, and 3 participants (7.3%) had extensive experience.

#### 3.2.1. Descriptive Statistics for Individual Items

All 12 Likert items received high levels of agreement, especially concerning curriculum inclusion (92.7%), stress reduction or improved focus (85.4%), facial scanning enhancing realism (80.5%), or ease of system usage (80.5%), according to Table 1.

#### 3.2.2. Associations Regarding Previous Experience with VR or Haptic Simulators

Results shown in Table 2 show that participants had no significant differences regarding previous experience with VR or haptic simulators according to age, gender or study year.

#### 3.2.3. Associations Regarding Survey Items

Regarding the perceived improvement in confidence in approaching clinical cases following training on the digital twin patient model, differences were not significant after controlling for FDR, across both gender (adjusted *p* = 0.216) and study year (adjusted *p* = 0.150). In exploratory, unadjusted terms, strong agreement was more frequent among male respondents (82.4%) than among female respondents (50.0%), whereas half of the female respondents (50.0%) and 11.8% of the male respondents selected “agree” (unadjusted *p* = 0.018).

Similarly, strong agreement was most frequent among fourth-year respondents (80.0%), compared with 33.3% of fifth-year respondents, while “agree” was selected most frequently by fifth-year respondents than fourth-year respondents (66.7% vs. 15%; unadjusted *p* = 0.025). These are exploratory patterns that did not persist after correction for multiple comparisons and require checking in future work, as detailed in Table 3.

Regarding agreement with the statement that digital twin simulation can complement conventional training, statistically significant differences were observed only for study year (adjusted *p* = 0.036) after controlling for FDR, without any significant differences across age (adjusted *p* = 0.160). In exploratory, unadjusted terms, strong agreement was most frequent among respondents aged 22 years, without any significant differences between groups (according to applied Z-tests) (92.9%; unadjusted *p* = 0.044).

Strong agreement was expressed by 90.0% of fourth-year respondents, compared with 41.7% of fifth-year respondents, while “agree” was selected most frequently by fifth-year respondents (58.3%) than fourth-year respondents (5%). These are exploratory patterns that require checking in future work, as detailed in Table 4.

## 4. Discussion

At the physical level, the developed digital twin patient model may enable more detailed exploration of dental pathology, alongside case rehearsal and clinical planning; it may further serve as an eco-friendly, reusable training tool—a potential, though here untested, contribution to sustainable medical education. At the psychological level, repeatable, risk-free rehearsal may support the gradual development of clinical confidence and promote learning in a gamified environment, which has been shown to improve engagement and retention [18,19,20,21,22]. Potential advantages that were not directly measured in this study, and that warrant future investigation, may include personalization, long-term cost efficiency, repeatability, and patient safety through risk-free training. Nevertheless, some limitations must be acknowledged. The initial investment required for haptic simulators is high, which may hinder widespread adoption [6].

### 4.1. Positioning the Multimodal Digital Twin Relative to Earlier Patient-Centred VR Training

Patient-centred VR training has previously relied on single-modality replicas of the patient, most notably intraoral scan-based dental arches [11]. The present workflow extends this concept by integrating three imaging modalities—intraoral, facial, and CBCT—into a single, haptic-simulator-compatible model, reproducing not only the dentition but the craniofacial skeleton and facial soft tissues of one identified patient. This multimodal integration, rather than any claim of real-time synchronisation, is what we intend the term “digital twin” to capture in the present, static implementation.

### 4.2. Findings from the Feasibility Pilot: Perceived Educational Value, Usability, and Associated Factors

Consistent with the ongoing digital transformation of dental education reported elsewhere [23,24,25], survey outcomes indicated a high level of acceptability and predominantly favourable perceptions of the PC-VR module among participants, with Likert ratings of 4–5 predominating across items.

#### 4.2.1. Positive Support for Curricular Integration

Item #11 (support for curricular inclusion) received the highest level of agreement among all items (Table 1), reflecting strong perceived readiness for integration into formal dental training and echoing the broader trend toward VR–haptic adoption in dental curricula [1,2,6,26,27,28]. Students similarly perceived the model as complementary to, rather than a replacement for, conventional training (Item #12; Table 1), consistent with the role simulation-based technologies are reported to play in narrowing the preclinical-to-clinical gap [1,6,8]. This aligns with broader reports that dental schools are increasingly adopting or planning VR-based curricular modules [29,30,31,32,33], including an international survey of implementation trends [10], and with calls for greater integration of advanced technologies in patient-centred dental education [9,34,35].

#### 4.2.2. Psychological Impact and Confidence Boost

High agreement with Item #5 (reduced stress, improved focus) and Item #10 (user-friendliness) (Table 1) is consistent with a perceived safe, risk-free training environment, an important consideration given that dental training inherently involves invasive and irreversible procedures on patients [11]. Repeated, risk-free practice of irreversible interventions (Item #4) and improved perceived confidence in approaching clinical cases (Item #7) are in line with prior reports identifying anxiety reduction and enhanced perceived preparedness among the principal reported benefits of VR–haptic implementation [10,19,36,37,38,39], and with the broader recognition of psychological safety as central to simulation-based learning [10,12,40,41,42].

#### 4.2.3. Anatomical Fidelity and Patient-Centred Cognitive Engagement

Perceived anatomical fidelity was high, with facial-scan integration (Item #8) and overall anatomical representation (Item #1) both rated favourably; the multimodal dataset provides a patient-specific reference framework not available with conventional phantom-head simulators [43]. Whether this patient-specific fidelity translates into a measurable learning advantage over conventional phantom heads remains an open question, to be addressed through dedicated comparative studies, consistent with reports linking high-fidelity virtual representation to learner engagement and immersion [44,45,46], and with broader literature associating VR/AI-enabled simulation with realistic case rendering, immediate feedback, and perceived clinical preparedness [45,46]. This fidelity extended to pedagogical engagement beyond anatomy: haptic simulation has been shown to support early restorative training [47], and improved perceived understanding of dental morphology (Item #2) is consistent with reports that three-dimensional manipulation aids the translation of theory into practice [48]. The personalized nature of the model was further associated with an increased perceived sense of responsibility toward patient-centred care (Item #6) [49], in line with the recognized contribution of mixed-reality haptic simulation to cognitive development, clinical reasoning, and exposure to anatomical variability [47,48,49].

#### 4.2.4. Usability and Perceived Potential for Standardized Assessment

High perceived usability (Item #10) may suggest that the system was perceived by students as easy to learn; no objective usability metric (e.g., time-on-task or error rate) was collected to confirm this. Usability is nonetheless widely regarded as a prerequisite for the scalability and standardization of simulation-based assessment [8,9,30], and ease of adoption is reported as a key determinant of successful VR–haptic implementation [10,50,51,52,53,54,55]. The favourable usability outcomes observed in the present study may be worth exploring in future multi-centre studies using objective usability measures, though this cannot be inferred from perception data alone.

#### 4.2.5. Interpretation of Statistical Findings and Associations

No significant associations were found between prior VR/haptic experience and age, gender, or study year (*p* = 0.068–0.627), suggesting a broadly homogeneous baseline exposure, consistent with the heterogeneity reported elsewhere in the literature [19,48]; prior experience may nonetheless shape performance dynamics in virtual environments [56], and VR-based pre-training has been reported to enhance skill acquisition in comparable tasks [36]. After controlling for multiple comparisons (FDR), only study year remained significantly associated with perceived complementarity with conventional training (adjusted *p* = 0.036); the confidence-related item showed no significant association with either gender or study year after adjustment (adjusted *p* = 0.216 and *p* = 0.150, respectively), nor did age show a significant association with the complementarity item (adjusted *p* = 0.160). In exploratory, unadjusted analyses, male and fourth-year respondents tended to be more frequently associated with the strongly agree response on the confidence-related item, and younger and fourth-year respondents with the strongly agree response on the complementarity item; as age and study year are closely correlated in this cohort, these exploratory patterns should not be interpreted as independent demographic influences and require confirmation in larger samples. These patterns are broadly consistent with reports that earlier-stage learners may hold more favourable attitudes toward digital and virtual learning environments [56,57], and with the recognized role of VR–haptic systems in providing structured, low-risk skill-acquisition environments [6,7,30,58,59,60,61]; given the exploratory nature of these comparisons and the small subgroup sizes involved, they should be read as hypothesis-generating rather than confirmatory (see Limitations).

### 4.3. Open Questions and Directions for Future Validation

The model’s lowest-rated item concerned its incomplete reproduction of dynamic, patient-specific function relative to conventional phantom-head simulators (Item #3, Table 1), indicating that they retain value alongside the proposed method and supporting a hybrid training approach [1,6,7,10,28,29]. This reflects the model’s present static character; the transition toward a dynamically synchronized digital twin—incorporating soft-tissue, saliva, and facial-animation dynamics—remains a recognized challenge for current haptic systems generally [10,31,49,52,53,54,55], and is identified here as a direction for future development rather than a feature of the present implementation. More broadly, this feasibility study cannot establish whether perceived benefits translate into measurable skill acquisition, confidence, or clinical performance: it involved no comparator group, no pre-workshop baseline, no objective usability or performance measure, and no evidence of transfer to patient care, and relied on an exploratory, non-validated instrument administered at a single time point (see Limitations). These questions define the priorities for the next phase of this research.

### 4.4. General Limitations and Future Directions

The model itself carries technical limitations that define priorities for future development. As implemented, it constitutes a static, multimodally integrated reconstruction without continuous or bidirectional linkage to the physical patient. Dynamic fidelity remains limited, as soft-tissue behaviour, tongue movement, saliva, and facial animation are not yet simulated; the transition from this static instantiation to a dynamically synchronized digital twin therefore remains an open challenge and represents a key direction for future development (Section 4.3). The software currently supports only voxel-based rendering and cannot assign distinct material densities to individual tissues, limiting its applicability to procedures such as endodontic treatment planning, although inclusion of the pulp chamber already allows instructors to assess preparation relative to pulpal proximity; future integration of tissue-specific density allocation and micro-CT-based pulp chamber segmentation would extend the range of procedures that can be simulated. The model also does not yet replicate patient–practitioner communication, an essential component of clinical training; future work could incorporate AI-driven virtual patients, conversational agents, and deepfake-based facial animation to address this gap, alongside expansion into comprehensive digital twin libraries and AI-driven assessment frameworks. A limitation of this study is the absence of a quantitative assessment of registration accuracy between the constituent datasets; fidelity claims are therefore qualitative and reflect feasibility rather than validated anatomical precision. Quantitative validation, using metrics such as root-mean-square surface deviation, is a priority for future work.

The questionnaire was purpose-built for this study and underwent only a content clarity review rather than formal psychometric validation; it should not be described as reliable, valid or reproducible in the psychometric sense. All 12 evaluative items were positively framed, several presupposing improvement or reduced stress, which may have introduced acquiescence bias, and administration on the same day as a single faculty-led workshop, while the technology and instructor were still novel, raises the possibility of social desirability and novelty effects that this design cannot disentangle from genuine educational benefit. The three thematic item groupings are descriptive rather than statistically validated domains, and all analyses were conducted at the individual item level. Subgroup comparisons by age, gender and study year were exploratory and not prespecified and involved small and in places sparse cells; the Benjamini–Hochberg correction was applied within each demographic family of 12 item-level comparisons, and no further adjustment was made across the three families, so that error across the full set of 36 comparisons is not fully controlled; age and study year are additionally closely correlated in this cohort and should not be interpreted as independent influences. This was, in addition, a single-timepoint, single-centre pilot study based on a single patient model, with participants recruited by convenience sampling; eligibility was additionally restricted to students who had passed the corresponding semester examination, so that the sample represents students meeting the faculty’s standard academic progression requirements rather than the full student population; 9 of 50 eligible students did not respond, and the resulting sample (*n* = 41) limits the generalizability of these findings to other cohorts or institutions. No comparator group trained by conventional methods, pre-workshop baseline measure, objective usability assessment, or performance or clinical-transfer outcome was included; claims regarding confidence, stress reduction, skill acquisition, usability, standardization, cost efficiency, sustainability and clinical preparedness are therefore based on perception data alone and should not be interpreted as demonstrated effects. The clustering of responses toward strong agreement further suggests a possible ceiling effect, and given the sampling strategy, the study should be regarded as exploratory and its findings as preliminary.

Future research should prioritize larger, multi-institutional and longitudinal cohorts; psychometric validation of the survey instrument, including reverse-scored items and internal-consistency testing; blinded and delayed survey administration with a comparison group; and validation of skill transfer through objective performance assessment in real clinical settings or comparisons between faculty and student perceptions.

## 5. Conclusions

The proposed method for generating a digital twin patient model integrated into an MR haptic-based dental simulator was perceived by students as accessible, enabling them to visualize and interact with a patient representation while rehearsing clinical procedures in a simulated environment. In this single-centre pilot study, students surveyed on the same day, following a single workshop, expressed positive perceptions of the approach and the model, and strong support for its curricular integration, though these findings reflect self-reported perceptions rather than demonstrated performance or clinical outcomes. After adjustment for multiple comparisons, only study year remained significantly associated with perceived complementarity with conventional training, favouring fourth-year students; the remaining demographic associations were exploratory in nature and should be regarded as patterns that need checking in future work. The digital twin patient model concept may hold potential as an educational tool across dental disciplines, pending validation through comparative, longitudinal and performance-based research.

## Figures and Tables

**Table 1:** Distribution of participants according to survey items referring to the proposed digital twin patient model.

Item Number (Nr., %)	Strongly Disagree	Disagree	Neutral	Agree	Strongly Agree
Item 1—The integrated digital twin patient model (intraoral, facial, and CBCT scans) provided a realistic representation of patient-specific anatomy	0 (0%)	0 (0%)	0 (0%)	9 (22%)	32 (78%)
Item 2—The alignment of hard and soft tissues in the digital twin patient model improved my understanding of clinical morphology	0 (0%)	0 (0%)	0 (0%)	11 (26.8%)	30 (73.2%)
Item 3—The digital twin simulation reproduced patient-specific conditions more effectively than conventional phantom-head simulators	0 (0%)	1 (2.4%)	5 (12.2%)	11 (26.8%)	24 (58.5%)
Item 4—Practicing with the digital twin patient model helped me prepare for irreversible procedures	0 (0%)	0 (0%)	4 (9.8%)	8 (19.5%)	29 (70.7%)
Item 5—The simulation environment allowed me to focus on procedural learning without the stress associated with treating real patients	0 (0%)	0 (0%)	0 (0%)	6 (14.6%)	35 (85.4%)
Item 6—Working on a personalized digital model increased my sense of responsibility towards patient-centered care	0 (0%)	1 (2.4%)	0 (0%)	11 (26.8%)	29 (70.7%)
Item 7—Using the digital twin patient model improved my confidence in approaching clinical cases	0 (0%)	0 (0%)	1 (2.4%)	14 (34.1%)	26 (63.4%)
Item 8—The integration of facial scans enhanced the realism of case documentation and treatment rehearsal	0 (0%)	0 (0%)	0 (0%)	8 (19.5%)	33 (80.5%)
Item 9—Compared to conventional phantom-head simulators, the patient-centered VR (Virtual Reality) environment provided a more meaningful learning experience	0 (0%)	0 (0%)	2 (4.9%)	8 (19.5%)	31 (75.6%)
Item 10—The proposed method was user-friendly	0 (0%)	0 (0%)	1 (2.4%)	7 (17.1%)	33 (80.5%)
Item 11—I would support the inclusion of digital twin patient model training in the dental curriculum	0 (0%)	0 (0%)	1 (2.4%)	2 (4.9%)	38 (92.7%)
Item 12—I believe digital twin simulations could complement, rather than replace, conventional phantom-head simulations	0 (0%)	0 (0%)	2 (4.9%)	9 (22%)	30 (73.2%)

**Table 2:** Distribution of participants according to demographic characteristics and previous experience with VR or haptic simulators.

Age (Nr., %)	None	Limited	Moderate	Extensive	*p* *
22 years	4 (28.6%)	7 (43.8%)	2 (25%)	1 (33.3%)	0.068
23 years	8 (57.1%)	3 (18.8%)	2 (25%)	0 (0%)	
24 years	1 (7.1%)	3 (18.8%)	4 (50%)	0 (0%)	
25 years	1 (7.1%)	3 (18.8%)	0 (0%)	2 (66.7%)	
Gender	None	Limited	Moderate	Extensive	*p* *
Female	7 (50%)	11 (68.8%)	5 (62.5%)	1 (33.3%)	0.627
Male	7 (50%)	5 (31.2%)	3 (37.5%)	2 (66.7%)	
Study year	None	Limited	Moderate	Extensive	*p* *
4th year	8 (57.1%)	8 (50%)	3 (37.5%)	1 (33.3%)	0.469
5th year	5 (35.7%)	4 (25%)	3 (37.5%)	0 (0%)	
6th year	1 (7.1%)	4 (25%)	2 (25%)	2 (66.7%)	

* Fisher’s Exact Test.

**Table 3:** Distribution of participants by gender, study year, and responses to Item 7 (improvement in confidence).

Gender	Neutral	Agree	Strongly Agree	*p* *
Female	0 (0%)	**12 (50.0%)**	**12 (50.0%)**	0.018 (Adjusted *p* = 0.216 **)
Male	1 (5.9%)	**2 (11.8%)**	**14 (82.4%)**	
Study year	Neutral	Agree	Strongly Agree	*p* *
4th year	1 (5.0%)	**3 (15.0%)**	**16 (80.0%)**	0.025 (Adjusted *p* = 0.150 **)
5th year	0 (0%)	**8 (66.7%)**	**4 (33.3%)**	
6th year	0 (0%)	3 (33.3%)	6 (66.7%)	

* Fisher’s Exact Test, ** Benjamini–Hochberg FDR corrected *p*-value. Percentages are calculated within each demographic group (row percentages). Values in bold indicate response categories that differed significantly between the groups compared, on post hoc z-tests of column proportions with Bonferroni correction.

**Table 4:** Distribution of participants by age, study year, and responses to Item 12 (complementing conventional training).

Age	Neutral	Agree	Strongly Agree	*p* *
22 years	1 (7.1%)	**0 (0%)**	**13 (92.9%)**	0.044 (Adjusted *p* = 0.160 **)
23 years	0 (0%)	5 (38.5%)	8 (61.5%)	
24 years	0 (0%)	3 (37.5%)	5 (62.5%)	
25 years	1 (16.7%)	1 (16.7%)	4 (66.7%)	
Study year	Neutral	Agree	Strongly Agree	p *
4th year	1 (5.0%)	**1 (5.0%)**	**18 (90.0%)**	0.003 (Adjusted *p* = 0.036 **)
5th year	0 (0%)	**7 (58.3%)**	**5 (41.7%)**	
6th year	1 (11.1%)	1 (11.1%)	7 (77.8%)	

* Fisher’s Exact Test, ** Benjamini–Hochberg FDR corrected *p*-value. Percentages are calculated within each demographic group (row percentages). Values in bold indicate response categories that differed significantly between the groups compared, on post hoc z-tests of column proportions with Bonferroni correction.

## Data Availability

The row-level dataset is available from the corresponding authors upon reasonable request, consistent with the confidentiality terms under which it was collected.

## Supplementary Materials

The following supporting information can be downloaded at: https://www.mdpi.com/article/10.3390/dj14100642/s1, The complete questionnaires, in English and in Romanian, are provided as Supplementary Materials S1 and S2.

## Abbreviations

The following abbreviations are used in this manuscript:

VR	Virtual Reality
MR	Mixed-reality
CBCT	Cone-Beam Computed Tomography
PC-VR	Patient-Centered Virtual Reality (concept)
AI	Artificial Intelligence
DICOM	Digital Imaging and Communications in Medicine
