# Supplementary material for: A Patient-Specific Digital Twin Model for Haptic-Based Dental Simulation: Development and Pilot Evaluation of Its Static Design
Source: Dent J (Basel). 2026 Oct 5;14(10):642. doi: 10.3390/dj14100642 (PMC13703461; doi:10.3390/dj14100642)
Supplement: Supplementary file 1 [file dentistry-14-00642-s001.zip › Supplementary_S1_Questionnaire_ENGLISH.pdf]

Supplementary Material S1

Study questionnaire (English version)

Section A. Perceptions of the digital twin patient model (items 1–12)

Items 1–12 were answered on a five-point response scale. All five points of the scale were verbally labelled: 1 = 'Strongly disagree', 2 = 'Disagree', 3 = 'Neutral', 4 = 'Agree', and 5 = 'Strongly agree'. All items in this section required a response.

1. The integrated digital twin patient model (intraoral, facial, and CBCT scans) provided a realistic representation of patient-specific anatomy.
2. The alignment of hard and soft tissues in the digital twin patient model improved my understanding of clinical morphology.
3. The digital twin simulation reproduced patient-specific conditions more effectively than conventional phantom-head simulators.
4. Practicing with the digital twin patient model helped me prepare for irreversible procedures.
5. The simulation environment allowed me to focus on procedural learning without the stress associated with treating real patients.
6. Working on a personalized digital model increased my sense of responsibility towards patient-centered care.
7. Using the digital twin patient model improved my confidence in approaching clinical cases.
8. The integration of facial scans enhanced the realism of case documentation and treatment rehearsal.
9. Compared to conventional phantom-head simulators, the patient-centered VR (Virtual Reality) environment provided a more meaningful learning experience.
10. The proposed method was user-friendly.
11. I would support the inclusion of digital twin patient model training in the dental curriculum.
12. I believe digital twin simulations could complement, rather than replace, conventional phantom-head simulations.

Section B. Respondent characteristics (items 13–16)

13. Please enter your age (in completed years; numbers only):
14. Please enter your gender:
    - ○ female
    - ○ male

- Other / I prefer not to answer

15. Year of study:
    - 1
    - 2
    - 3
    - 4
    - 5
    - 6
    - Dentist / Resident

16. Previous experience with VR or haptic simulators
    - None
    - Limited
    - Moderate
    - Extensive

Notes

All remaining items were required. The questionnaire was administered online and responses were collected anonymously.
