# Supplementary material for: A Patient-Specific Digital Twin Model for Haptic-Based Dental Simulation: Development and Pilot Evaluation of Its Static Design
Source: Dent J (Basel). 2026 Oct 5;14(10):642. doi: 10.3390/dj14100642 (PMC13703461; doi:10.3390/dj14100642)
Supplement: Supplementary file 1 [file dentistry-14-00642-s001.zip › Supplementary_S2_Questionnaire_ROMANIAN version.pdf]

Supplementary Material S2

Chestionarul studiului (versiunea în limba română)

Secțiunea A. Percepții privind „the digital twin patient model"/ „modelul digital twin (geaman) al pacientului" (itemii 1–12)

Itemii 1–12 au fost evaluați pe o scală de răspuns în cinci puncte. Toate cele cinci puncte ale scalei au fost etichetate verbal: 1 = „Dezacord total", 2 = „Dezacord", 3 = „Neutru", 4 = „Acord" și 5 = „Acord total". Toți itemii din această secțiune au necesitat un răspuns.

1. Geamănul digital integrat (scanări intraorale, faciale și CBCT) a oferit o reprezentare realistă a anatomiei specifice pacientului.
2. Alinierea țesuturilor dure și moi în cadrul „digital twin patient model" mi-a îmbunătățit înțelegerea morfologiei clinice.
3. Simularea cu „digital twin patient model" a reprodus condițiile specifice pacientului mai eficient decât manechinele convenționale.
4. Exersarea cu „digital twin patient model" m-a ajutat să mă pregătesc pentru proceduri ireversibile.
5. Mediul de simulare mi-a permis să mă concentrez pe învățarea procedurală fără stresul asociat tratării pacienților reali.
6. Lucrul pe un model digital personalizat mi-a crescut simțul responsabilității față de îngrijirea centrată pe pacient.
7. Utilizarea „digital twin patient model" mi-a îmbunătățit încrederea în abordarea cazurilor clinice.
8. Integrarea scanărilor faciale a crescut realismul documentării cazului și al repetiției tratamentului.
9. În comparație cu simulatoarele generice de tip cap-fantomă, mediul VR centrat pe pacient a oferit o experiență de învățare mai relevantă.
10. Metoda propusă a fost ușor de utilizat.
11. Aș susține includerea instruirii bazate pe „digital twin patient model" în curricula stomatologică.
12. Consider că simulările cu „digital twin patient model" ar putea completa, nu înlocui, metodele tradiționale de pregătire preclinică.

Secțiunea B. Caracteristicile respondenților (itemii 13–16)

13. Vă rugăm introduceți vârsta (în ani împliniți; doar cifre):
14. Vă rugăm indicați genul:
    - ○ feminin

- masculin
- Altul / Prefer să nu răspund

15. Anul de studiu:
    - 1
    - 2
    - 3
    - 4
    - 5
    - 6
    - Medic dentist / Rezident

16. Experiență anterioară cu simulatoare VR sau haptice
    - Fără
    - Limitată
    - Moderată
    - Extinsă

Note

Toți itemii rămași au fost obligatorii. Chestionarul a fost administrat online, iar răspunsurile au fost colectate anonim.

The wording of the items above reflects the text originally administered to students; only the descriptive terminology used in the manuscript has been harmonised.
